# Differences in endosomal Rab gene expression between positive and negative COVID-19 patients

**DOI:** 10.1186/s13104-022-06144-7

**Published:** 2022-07-15

**Authors:** Nur Atik, Farruqi Wirawan, Riezki Amalia, Astrid Feinisa Khairani, Gita Widya Pradini

**Affiliations:** 1 Department of Biomedical Sciences, Faculty of Medicine, Universitas Padjadjaran, Bandung, West Java 40161 Indonesia; 2 Faculty of Medicine, Universitas Padjadjaran, Bandung, West Java Indonesia; 3 Department of Pharmacology and Clinical Pharmacy, Faculty of Pharmacy, Universitas Padjadjaran, Bandung, West Java Indonesia

**Keywords:** Endocytic pathway, Rab5, Rab7, Rab11b, SARS CoV-2, Viral entry

## Abstract

**Objective:**

SARS CoV-2, the etiologic agent of coronavirus disease-2019 (COVID-19) is well-known to use ACE2 to begin internalization. Some viruses enter the host cell through the endocytosis process and involve some endocytosis proteins, such as the Rab family. However, the relationship between SARS CoV-2 infection with endocytic mRNA *RAB5, RAB7*, and *RAB11B* is unknown. This study aims to compare the expression of *RAB5, RAB7,* and *RAB11B* between positive and negative COVID-19 patient groups.

**Results:**

Both viral and human epithelial RNA Isolation and RT-PCR were performed from 249 samples*.* The genes expression was analysed using appropriate statistical tests. We found the Median (inter-quartile range/IQR) of *RAB5, RAB7,* and *RAB11B* expression among the COVID-19 patient group was 2.99 (1.88), 0.17 (0.47), 0.47 (1.49), and 1.60 (2.88), 1.05 (2.49), 1.10 (3.96) among control group respectively. We proceeded with Mann Whitney U Test and found that *RAB5* expression was significantly increased (*P* < 0.001), and *RAB7* and *RAB11B* expression was significantly decreased (*P* < 0.001 and *P* = 0.036) in the COVID-19 patient group compared to the control group. This first report showed significant differences in *RAB5, RAB7*, and *RAB11B* exist between COVID-19 positive and negative patients.

## Introduction

Coronavirus disease-2019 (COVID-19) is an infectious viral disease caused by a novel coronavirus called severe acute respiratory syndrome coronavirus 2 (SARS-CoV-2) [1–3]. In less than 3 months, the condition has already gained pandemic status from WHO [4]. This alarming rate of spread is supported by the fact that this disease can spread quickly from person to person by respiratory droplet, either directly from inhalation of the infected droplet or indirectly by touching a surface contaminated by this virus and then touching the face, mouth, or nose [3]. Together with SARS CoV and MERS CoV, which also caused an outbreak in 2002 and 2012, SARS CoV-2 can bring severe lung injury and respiratory pathologies [3, 5]. SARS CoV-2 is a single-strand positive sense enveloped RNA virus with genome arrangement as follows: 5′-orf1ab-Spike (S)-Envelope (E)-Membrane (M)-Nucleocapsid (N)-3′. ORF1ab is the first open reading frame that encodes either replicase polyprotein pp1a or pp1ab. This region cleaves proteolytically into 16 non-structural proteins (NSPs). On the other side, nucleocapsid protein, together with spike, envelope, and membrane protein are structural proteins [3, 6].

As an obligate intracellular parasite, viruses must deliver their genome to the nucleus of host cells to replicate and ultimately cause disease. Coronavirus entry, therefore, plays an integral role as it connects the viral receptor binding phase and viral fusion and uncoating phase of its life cycle [5, 7]. Most viruses utilize the host endocytic pathway to enter the cells [8]. Influenza A virus utilises this pathway to gain entry into its host cells. It has six steps to enter target cells that start with attachment to target cells and are followed by internalization into cellular compartments, trafficking through endosomal network, fusion, uncoating, and finally viral genome entry into the nucleus [9]. There are vast arrays of proteins to regulate endocytic pathways, and Rab proteins are one of them. Rab protein belongs to the Ras superfamily of Small GTPase. Rab5 is known to have a role in the early endosome. The Rab5 regulates the formation of clathrin-coated vesicles (CCV), the fusion of CCV with early endosomes, and homotypic fusion between early endosomes [10, 11]. Rab7 regulates the early endosome to late endosome by a process called Rab5 to Rab7 switch. It also regulates the endocytic process downstream of the late endosome, transported from the late endosome to the lysosome, lysosome biogenesis, and fusion of the late endosome with a lysosome. In addition to endocytosis, Rab7 is also known to have a role in several other cellular functions, like retrograde trafficking, phagocytosis, autophagy, mitophagy and apoptosis [10, 12]. Rab11b regulates the endocytic process in recycling endosomes [10]. Rab11b, alongside Rab11a and Rab25 are the three members of the Rab11 subfamily. Generally, the Rab11 subfamily plays a role in the plasma membrane recycling system and cell polarity, with each subfamily member having a specific role [13]. Rab11a plays an essential role in localising the intestinal apical protein, Rab11b in the recycling of transferrin, and Rab25 in the apical recycling endosome [13, 14].

It is well known that SARS CoV-2 Spike protein interacts with ACE2 in the receptor binding phase to begin internalization [15]. This is supported by the fact that leukaemia patients, which have increased ACE2 expression levels, have an increased risk of COVID-19 infection and more severe disease manifestation [16]. However, the differences in endosomal *RAB5, RAB7,* and *RAB11B* mRNA expression between positive and negative covid-19 patients are still unknown. Therefore, this study aims to compare the expression of *RAB5, RAB7,* and *RAB11B* between the COVID-19 positive and negative groups as categorized by the SARS CoV-2 *ORF1ab* and *N-gene* expression.

## Main text

### Materials and methods

#### RNA samples collection

This study used the stored biological material from 249 human nasopharyngeal swab samples collected in Jatinangor and Bandung, West Java, Indonesia, from May 2020 to October 2020. The samples were considered positive or negative according to the guidelines of the RT PCR kit. 199 and 50 COVID-19 positive and negative, respectively, stored biological materials were used.

#### RNA isolation

Total RNA Isolation was performed for all stored biological materials using Zybio Nucleic Acid Extraction Kit in four steps, including pretreatment, lysate, rinsing, and elution. First, a working solution was made by mixing 500 μL Isolation Reagent I, 4 μL Magnetic Beads Solution, and 15 μL Proteinase K. To make the lysate, 500 μL of the working solution was mixed well with 200 μL sample, lysed at 55 °C for 4 min, absorbed by magnetic separator for 1 min, and discarded the supernatant. The solution is then rinsed by adding 600 μL of Isolation Reagent II, mixed well, absorbed by a magnetic separator for 1 min, and discarded the supernatant. Finally, to elute the sample, 50 ~ 100 μL Elution Buffer was added.

#### RT-PCR of *ORF1ab* and *N-Gene*

RT-PCR for viral *ORF1ab* and *N-gene* RNA was performed using the Detection Kit for 2019 Novel Coronavirus (2019-nCoV) RNA, (PCR-Fluorescence Probing) from Da An Gene Co., Ltd. of Sun Yat-sen University. The temperature and time used for thermal cycling were as follows: 1 cycle of reverse transcription at 50 °C for 15 min, one cycle of DNA Polymerase activation at 95 °C for 15 min, and then 45 cycles of melt and anneal/extend at 95 °C and 60 °C for 15 s and 1 min each. We were run qPCR using LightCycler® 96 System, Roche.

#### RT-qPCR of *RAB5, RAB7*, and *RAB11B*

RT-PCR for *RAB5, RAB7,* and *RAB11B* were performed using Promega (GoTaq® 1-Step RT-qPCR System). *GAPDH* was used to normalize the Ct values of *RAB5, RAB7* and *RAB11B* in the quantification using the Modified 2-ΔΔCt (Livak) method [17]. Primers used for *RAB5, RAB7, RAB11B,* and *GAPDH* RT-PCR were as follows: primer ‘RAB5f’, 5′-TGG GAT ACA GCT GGT CAA GA-3′; primer ‘RAB5r’, 5′-GGA CTT GCT TGC CTC TGA AG-3′ [18]; primer ‘RAB7f’, 5′-AAG CCA CAA TAG GAG CTG AC-3′; primer ‘RAB7r’, 5′-CAA TCT TGT TTC CCA ACA CA-3′; primer ‘RAB11Bf’, 5′-AAC GAG TTC AAC CTG GAG AG-3′; primer ‘RAB11Br’, 5′-ATG ATG ACG ATG TTG CTG TC-3′ [19]; primer ‘GAPDHf’, 5′-GAA GGT GAA GGT CGG AGT C-3′; primer ‘GAPDHr’, 5′-GAA GAT GGT GAT GGG ATT TC-3′. Fluorescent DNA-binding dye, BRYT Green® Dye was used as a fluorescent detection system. Temperature and time used for thermal cycling were as follows: 1 cycle of reverse transcription at 37 °C for 15 min, 1 cycle of reverse transcriptase inactivation and GoTaq® DNA Polymerase activation at 95 °C for 10 min, and then 40 cycles of denaturation, annealing and data collection, and extension at 95 °C, 60 °C, and 72 °C for 10 s, 30 s, and 30 s each.

#### Data analysis

The expression of *ORF1ab, N-gene, RAB5, RAB7*, and *RAB11B* from each sample was analysed using statistical software IBM SPSS Statistics 25. First, the normality of data distribution was analysed using the Kolmogorov–Smirnov test. If the data is distributed normally, the difference between *RAB5, RAB7,* and *RAB11B* from the COVID-19 positive and negative groups will be analysed using an independent t-test. But if the data did not distribute normally, the test used will be the Mann–Whitney U test instead. A significance level of 0.05 is used for all statistical tests.

### Results

From 249 samples, some data were cut out during data cleaning due to invalid values. Some flaws in the preparation process of RNA isolate might be the cause. For the *RAB5* expression, 196 positive and 49 negative samples were available to be analysed. For *RAB7*, 190 positive and 48 negative samples were available, and for *RAB11B* 190 positive and 49 negative samples were available. Using Kolmogorov–Smirnov Test revealed that the distribution of *RAB5, RAB7,* and *RAB11B* expression was not normal, and the Mann–Whitney U test was performed to test the difference in the median value of *RAB5, RAB7,* and *RAB11B* between COVID-19 positive group and negative group.

We showed that the 196 positive subjects had a relatively similar median for both CT values SARS CoV-2 genes in RT PCR examination. The median values were 31.67 and 30.67 for ORF1ab and N genes, respectively (Table 1).

**Table 1:** Characteristics of viral CT Value in COVID-19 positive

Gene	Median	Inter-quartile range
ORF1ab	31.67	12.80
N-gene	30.67	13.06
IC	24.65	3.04

Data showed from 196 positive subjects

Beginning with the regulator of early endosome, we found that the median (inter-quartile range/IQR) of *RAB5* expression among the COVID-19 positive group was 2.99 (1.88) and 1.60 (2.88) among the negative group. As expected, the COVID-19 positive group reported a significant increase in *RAB5* expression than the negative group (*P* < 0.001) (Table 2).

**Table 2:** Difference of *RAB5, RAB7,* and *RAB11B* expression between COVID-19 positive and negative patients

Gene	Group (n)	Median	IQR	*P-*value
*RAB5*	Positive (196)	2.99	1.88	(*P* < 0.001)
	Negative (49)	1.60	2.88	
*RAB7*	Positive (190)	0.17	0.47	(*P* < 0.001)
	Negative (48)	1.05	2.49	
*RAB11B*	Positive (190)	0.47	1.49	(*P* = 0.036)
	Negative (49)	1.10	3.96	

Due to either undetected or out of range gene expression, 3 *ORF1ab*, 5N*-gene*, 4 *RAB5*, 11 *RAB7*, and 10 *RAB11B* expression data were cut out. Double measurement was performed to obtain Ct values of each RAB mRNA

Further, we then proceed with *RAB7* and *RAB11B* analysis to determine the difference between late and recycling endosomes between SARS CoV-2 positive and negative patients. We found that the median (IQR) of *RAB7* expression among the COVID-19 positive group was 0.17 (0.47) and 1.05 (2.49) among the negative group. Strikingly, COVID-19 positive group reported a significant decrease in *RAB7* expression than the negative group (*P* < 0.001). The same also goes for *RAB11B*. COVID-19 positive group reported significant decrease in *RAB11B* expression than negative group (*P* = 0.036), from 1.10 to 0.47 (Table 2).

### Discussion

**Fig. 1:**
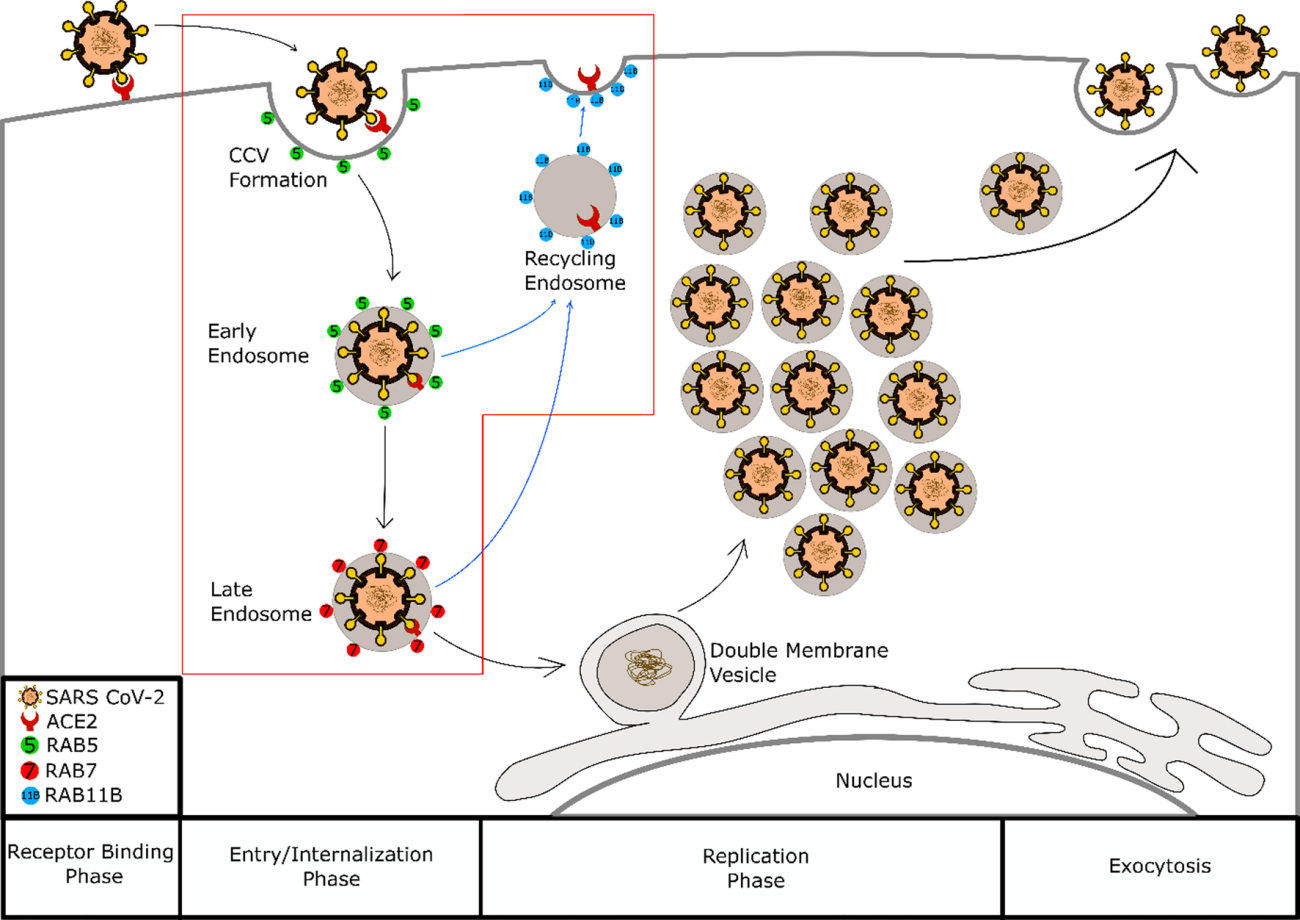
Our proposed model shows that Rab5, Rab7, and Rab11b play a role in the endocytosis of SARS CoV-2 to host cells. The black arrow indicates the viral lifecycle pathway, and the blue arrow indicates the recycling pathway for the viral receptor and endosome membrane

This study showed *RAB5* expression was significantly increased in COVID-19 patients, as proved by Mann–Whitney U Test. Rab5 is known to have an extensive function in the early endocytic pathway. It participates in forming clathrin-coated vesicles, the fusion of clathrin-coated vesicles with early endosomes, and homotypic fusion between early endosomes [10, 11]. Following the theory, this study's findings predictably show that SARS CoV-2 entry into host cells might be utilizing host early endosome and provide a basis for the development of Rab5 antagonist as an anti-SARS CoV-2 drug (Fig. 1).

On the other side, the decreased expression of *RAB7* in COVID-19 patients was not so easily predicted. In the endocytic pathway, Rab5 to Rab7 switch is a hallmark process in maturing the early endosome to the late endosome [10–12, 20, 21]. At a glance, this could explain the decreased expression of *RAB7* in COVID-19 patients with the assumption that the endosomes of infected cells are still in the early endosome phase and not yet matured into late endosomes. DNA damage is another possible explanation for the decreased expression of *RAB7* and *RAB11B*, as a previous study shows that SARS CoV-2 is known to induce a DNA damage response in Vero E6 cells [22].

The SARS CoV-2 fusion and uncoating and cytosol penetration phase that happens in early endosomes is another possible explanation for decreased expression of *RAB7* together with an increase of *RAB5* expression in COVID-19 patients*.* Viral particles entry into the cytosol could happen in various locations within the endocytic pathway, from the early endosome, late endosome, lysosome, and macropinosome, to the endoplasmic reticulum [7]. Viral particles entry into the cytosol of coronavirus is proposed to take place following endosomal maturation [23]. In certain circumstances where furin-like protease activates the virus, entry to cytosol in early endosome can also happen as active furin is known to present in early endosome (Fig. 1) [23, 24]. Interestingly, previous studies show that SARS CoV-2 has a furin cleavage site in its spike protein [25, 26].

This study cannot justify when the Rab5 to Rab7 switch happens, and so we still have a missing link between the decreased *RAB7* expression and Rab5 to Rab7 switching. Using DNA Damage theory, we also cannot find why the damage occurs only in *RAB7* and *RAB11B* genes but not in *RAB5*. Even the viral fusion in early endosome theory fails to explain why *RAB7* expression decreased, either unchanged. To further elucidate this study result, in the near future, we will perform in vitro study to ensure that those endocytic proteins are really involved in SARS CoV-2 endocytic uptake by creating *RAB5, RAB7*, and *RAB11B* knockdown epithelial cells and infecting them with SARS CoV-2.

Finally, we conclude that *RAB5* expression was significantly increased and *RAB7* and *RAB11B* expression was significantly decreased in the COVID-19 positive group compared to the negative group. Rab5 to Rab7 switch and DNA damage might explain this difference in *RAB5* and *RAB7* expression. DNA damage might explain the decreased expression of *RAB11B*. This result also shows a possibility that the SARS CoV-2 fusion and uncoating phase happens in an early endosome. This result can be used as a basis for developing a Rab5 antagonist that can be used as an anti-SARS CoV-2 drug.

## Limitation

This study cannot perform the protein detection method to observe the expression of Rab5, Rab7, and Rab11b due to the minuscule amount of each swab sample. We also could not show the specific reason to explain the decreased expression of *RAB7, and RAB11B,* as each of the theories still left some questions unanswered.

## Data Availability

The data are available from the corresponding author upon request.

## Abbreviations

COVID-19: Coronavirus disease-2019
ACE2: Angiotensin-converting enzyme 2
IQR: Inter-quartile range
SARS-CoV-2: Severe acute respiratory syndrome coronavirus 2
NSP: Non-structural proteins
CCV: Clathrin-coated vesicles
